# BNIP3 upregulation via stimulation of ERK and JNK activity is required for the protection of keratinocytes from UVB-induced apoptosis

**DOI:** 10.1038/cddis.2017.4

**Published:** 2017-02-02

**Authors:** Mariko Moriyama, Hiroyuki Moriyama, Junki Uda, Hirokazu Kubo, Yuka Nakajima, Arisa Goto, Takashi Morita, Takao Hayakawa

**Affiliations:** 1 Pharmaceutical Research and Technology Institute, Kindai University, Higashi-Osaka, Osaka, Japan

## Abstract

The human skin has an important role in barrier function. Ultraviolet rays (UV) from sunlight exposure can cause cell apoptosis in the skin epidermis, resulting in the disruption of the barrier. Previously, we have demonstrated that BNIP3 stimulates autophagy in epidermal keratinocytes and has a protective effect in these cells upon UVB irradiation. In this study, we found that the accumulation of reactive oxygen species (ROS) by UVB irradiation was sufficient to trigger the activation of JNK and ERK mitogen-activated protein kinase (MAPK) in human primary epidermal keratinocytes. In turn, activated JNK and ERK MAPK mediated the upregulation of BNIP3 expression. Treatment with an antioxidant reagent or a specific inhibitor of MAPK, U0126, and a JNK inhibitor significantly attenuated the expression of BNIP3 triggered by UVB, followed by the induction of cell death by apoptosis. Furthermore, UVB-induced apoptosis was significantly stimulated by chloroquine or bafilomycin A1, an inhibitor of autophagy. Moreover, BNIP3 was required for the degradation of dysfunctional mitochondria upon UVB irradiation. These data clearly indicated that BNIP3-induced autophagy, which occurs via UVB-generated ROS-mediated JNK and ERK MAPK activation, has a crucial role in the protection of the skin epidermis against UVB irradiation.

The stratified epithelium of the skin acts as a physical barrier against pathogens, toxins, and harmful irradiation. Epidermal keratinocytes are continuously exposed to environmental stresses including ultraviolet (UV) rays, which can cause DNA damage. Apoptosis serves as a protective mechanism by eliminating cells with damaged organelles, proteins, and/or DNA to reduce the risk of tumor formation; on the other hand, excessive apoptosis is associated with disruption of epidermal barrier function and inflammation of the skin. However, the molecular mechanisms whereby the cells maintain the balance between survival and cell death in response to stress conditions in order to maintain skin homeostasis is not completely understood.

We have previously shown that BCL2 and adenovirus E1B 19-kDa interacting protein 3 (BNIP3) is upregulated by UVB irradiation and is essential for the protection of keratinocytes from UVB-induced apoptosis. Furthermore, we also found that UVB stress induces autophagy and that knockdown of BNIP3 expression is sufficient to suppress this response.^1^ These data led us to speculate that the autophagy induced by BNIP3 might serve an anti-apoptotic function.

BNIP3 is a single-pass transmembrane protein located in the outer mitochondrial membrane. BNIP3 was first classified as a pro-apoptotic factor because it possesses a conserved BH3 domain that is essential for its pro-apoptotic activity and for heterodimerization with anti-apoptosis proteins.^2^ Recently, it has been reported that BNIP3 can induce apoptosis, necrosis, or autophagy depending on the cellular context.^3^ For example, hypoxia-induced BNIP3 expression in cardiomyocytes has been reported to correlate with apoptotic cell death.^4^ In contrast, it was also reported that BNIP3-mediated cell death is independent of Apaf-1, caspase activation, cytochrome *c* release, and nuclear translocation of apoptosis-inducing factors in MCF-7 and HeLa cells, indicating that BNIP3-induced necrosis-like cell death.^5^ Many studies have reported that BNIP3 induces autophagy; however, whether this leads to cell death or survival is controversial, as the induction of autophagy by BNIP3 has a protective effect in some conditions, whereas in others it is associated with autophagic cell death.^6, 7, 8^

Autophagy was described based on its ultrastructural feature of double-membraned structures that surrounded the cytoplasm and organelles in cells, known as autophagosomes.^9^ Autophagy is an evolutionarily conserved catabolic program by which cytoplasmic material and intracellular organelles are engulfed in autophagosomes, ultimately resulting in their degradation by the lysosomes. Although excessive autophagy has been shown to cause autophagic cell death, it is primarily a protective process for the cell. As a consequence of the activation of autophagy in response to various stresses including starvation, hypoxia, or changing nutrient conditions, the elimination of intracellular aggregates and damaged organelles is enhanced, thereby promoting survival. Recently, genotoxic stress has also been reported to induce autophagy, resulting in cytoprotection in multiple cell types.^10, 11^ For example, stress-causing DNA damage can be induced by several factors including chemical substances, ionizing radiation, reactive oxygen species (ROS), and UV irradiation.

Among UV radiation, UVB wavelengths (280–315 nm) penetrate only into the epidermis of the skin. However, UVB is the most energetic and causes skin disorders such as formation of sunburn cells.^12^ UVB is known to activate several signaling pathways including the p53, hypoxia-inducible factor (HIF), and mitogen-activated protein kinase (MAPK) signaling pathways. MAPK is a highly conserved family of serine/threonine protein kinases involved in a variety of fundamental cellular processes such as proliferation, differentiation, stress response, apoptosis, and survival. The conventional MAPK pathway consists of three components in mammals: the extracellular signal-regulated kinase (Erk), the c-Jun N-terminal kinase (JNK), and the p38 kinase pathways. It has been demonstrated that UVB activation of multiple cytokine and growth factor cell surface receptors including epidermal growth factor receptor (EGFR) and tumor necrosis factor-*α* receptor leads to the stimulation of these MAPK signaling transduction pathways. UVB irradiation has additionally been reported to result in the generation of ROS, which are also implicated in activating MAPK signaling pathways.^13, 14^

In this study, we focus on the molecular mechanism by which BNIP3 functions as a survival factor in response to UVB irradiation. Our data demonstrated that the ROS accumulation mediated by UVB irradiation is sufficient to trigger the activation of JNK and ERK MAPK in human primary epidermal keratinocytes (HPEKs), which in turn upregulated BNIP3 expression. Furthermore, we also found that inhibitors of autophagy significantly stimulated UVB-induced apoptosis. These data clearly indicated that BNIP3-induced autophagy occurs via UVB-generated ROS-mediated JNK and ERK MAPK activation and has a crucial role in the protection of the skin epidermis against UVB irradiation.

## Results

### UVB induces BNIP3 via ROS accumulation, protecting keratinocytes from apoptosis

**Figure 1:**
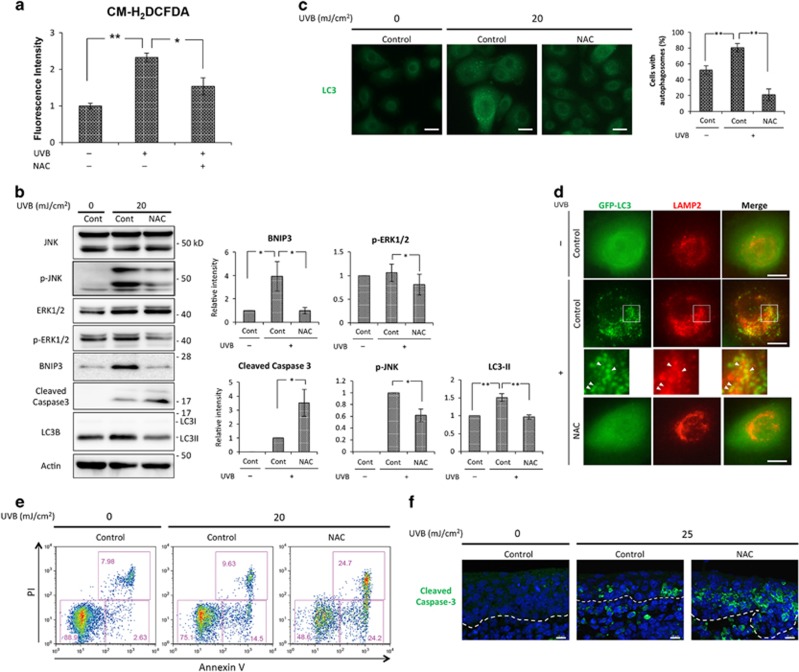
**BNIP3 expression is regulated by ROS in response to UVB exposure.** (**a**) HPEKs were irradiated with 20 mJ/cm^2^ UVB. NAC (5 mM) was added as an antioxidant 1 h before UVB exposure. Following irradiation, ROS levels were measured using a microplate reader. The data represent the average of relative fluorescence intensities from five independent experiments±S.E. **P<0.01. *P<0.05. (**b–e**) HPEK cells were exposed to UVB radiation (20 mJ/cm^2^) and incubated for 4 h. (**b**) The extracted proteins were then immunoblotted with the indicated antibodies. Graphs indicate relative band intensities as determined by ImageJ software and plotted as the means of seven independent experiments. **P<0.01. *P<0.05. Cont, control. (**c**) Fluorescence staining of endogenous LC3 expression (green) in HPEK cells is shown. Scale bars, 20 *μ*m. The graph indicates the percentages of cells with autophagosomes. Cells with more than five puncta were quantified and presented as the means of three independent experiments±S.E. In each experiment, 100–150 cells were analyzed. (**d**) Fluorescence staining of EGFP-LC3 expression (green) and LAMP2 (red) in HPEK cells is shown. The arrowheads mark the colocalization of the two proteins. Scale bars, 10 *μ*m. (**e**) HPEKs were stained with annexin V-Alexa 488 and propidium iodide (PI). Early apoptotic cells and late apoptotic/necrotic cells are defined by the annexin V^+^, PI^-^ and annexin V^+^, PI^+^ population, respectively. (**f**) The dorsal skin of a 14.5-dpc embryonic mouse was irradiated with 25 mJ/cm^2^ UVB and cultured on Millicell culture inserts for 12 h. Following organ culture, the skin sections were processed for anti-cleaved caspase 3 immunostaining (green). The blue signals indicate nuclear staining. The dotted lines indicate the boundary between the epidermis and the dermis. Scale bars, 10 *μ*m

As previously demonstrated, BNIP3 is upregulated and has a protective effect on epidermal keratinocytes upon UVB irradiation (Supplementary Figure S1).^1^ In addition, UVB irradiation induces autophagy, which is significantly attenuated by knockdown of BNIP3 expression.^1^ To reveal the molecular mechanism by which BNIP3 expression is activated by UVB irradiation, we first focused on ROS because previous studies suggested that ROS are accumulated following UVB exposure.^15, 16^ Consistent with the previous reports, we found that ROS production significantly increased when human primary epidermal keratinocytes (HPEKs) were irradiated by UVB (Figure 1a). We also determined that *N*-acetyl cysteine (NAC), which has been widely used as an antioxidant, significantly attenuated the UVB-induced ROS production (Figure 1a). As in our prior results, UVB irradiation upregulated BNIP3 expression and induced apoptosis as measured by the upregulation of the cleaved form of caspase 3 and the increase in both Annexin-V^+^PI^-^ (early apoptotic) and Annexin-V^+^PI^+^ (late apoptotic) cells (Figures 1b and e). Notably, UV-induced BNIP3 expression was significantly downregulated by NAC treatment, which was concomitant with downmodulation of the phosphorylated forms of JNK and ERK1/2 (Figure 1b). In addition, the autophagosome formation, detected by the upregulation of microtubule-associated protein 1A/1B-light chain 3 (LC3)-II (Figure 1b), the increase in LC3 puncta (Figure 1c), and the colocalization of GFP-LC3 puncta and lysosome (LAMP2 positive; Figure 1d), induced by UVB exposure was attenuated by NAC treatment, accompanied by BNIP3 expression downregulation (Figures 1b–d). Furthermore, western blot analysis against cleaved caspase 3 and flow cytometry analysis of annexin-V-PI staining revealed that apoptosis was increased when HPEKs were treated with NAC (Figures 1b and e). In accordance with these results, organ-cultured skin from e14.5 mouse embryos revealed that UVB-induced apoptosis was significantly increased when the skin was treated with NAC (Figure 1f). Together, these data indicate that ROS accumulation upon UVB treatment is involved in BNIP3 expression, which is required for the protection of keratinocytes from UVB-induced apoptosis. In addition, these findings suggested that ROS might also regulate JNK and ERK1/2 MAPK.

### UVB activates JNK and ERK1/2 MAPK signaling

**Figure 2:**
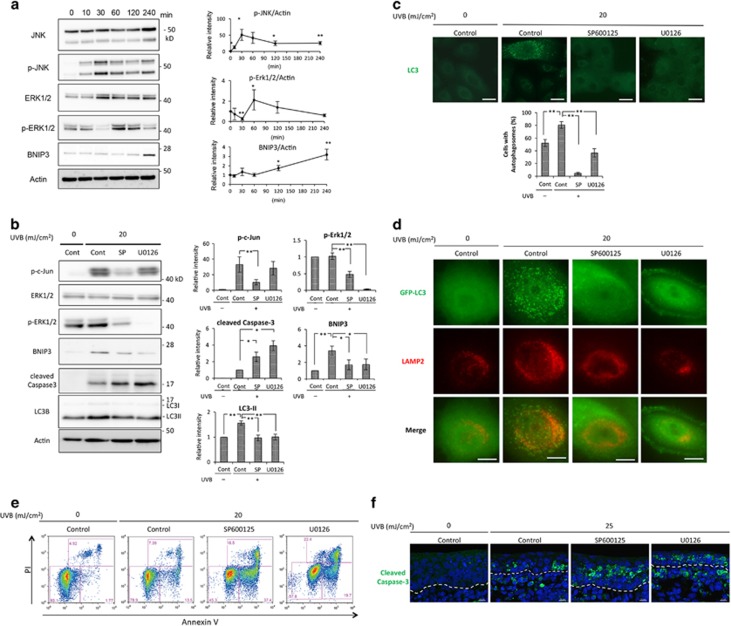
BNIP3 expression is regulated by ERK1/2 and JNK MAPK in response to UVB exposure. (**a**) HPEK cells were exposed to UVB radiation (20 mJ/cm^2^) and incubated for the indicated times. The extracted proteins were then immunoblotted with the indicated antibodies. Graphs indicate the relative band intensities as determined by ImageJ software and plotted as the means±S.E. of seven independent experiments. **P<0.01. *P<0.05. (**b–e**) HPEK cells were exposed to UVB radiation (20 mJ/cm^2^) and incubated for 4 h. SP600125 (10 *μ*M) and U0126 (10 *μ*M) were added 1 h before UVB exposure. (**b**) The extracted proteins were then immunoblotted with the indicated antibodies. Graphs indicate the relative band intensities as determined by ImageJ software and plotted as the means of eight independent experiments. **P<0.01. *P<0.05. (**c**) Fluorescence staining of endogenous LC3 expression (green) in HPEK cells is shown. Scale bars, 20 *μ*m. The graph indicates the percentages of cells with autophagosomes. Cells with more than five puncta were quantified and presented as the means of three independent experiments±SE. In each experiment, 100–150 cells were analyzed. (**d**) Fluorescence staining of EGFP-LC3 expression (green) and LAMP2 (red) in HPEK cells is shown. Scale bars, 10 *μ*m. (**e**) HPEKs were stained with annexin V-Alexa 488 and propidium iodide (PI). (**f**) The dorsal skin of a 14.5-dpc embryonic mouse was irradiated with 25 mJ/cm^2^ UVB, and cultured on Millicell culture inserts for 12 h. SP600125 (10 *μ*M) and U0126 (10 *μ*M) were added 1 h before UVB exposure. Following organ culture, the skin sections were processed for anti-cleaved caspase 3 immunostaining (green). The blue signals indicate nuclear staining. The dotted lines indicate the boundary between the epidermis and the dermis. Scale bars, 10 *μ*m

As it has been reported that JNK and ERK1/2 are responsible for UVB-induced apoptosis,^15, 17^ we studied the levels of JNK and ERK1/2 phosphorylation in keratinocytes. As shown in Figure 2a, western blot analysis revealed that the level of JNK phosphorylation in HPEKs started to increase significantly in the 0.5 h immediately following UVB irradiation (20 mJ/cm^2^). Phosphorylation of ERK1/2, on the other hand, was significantly decreased at 0.5 h after irradiation as previously reported.^18^ Although it showed an increase at 1 h, it subsequently returned to basal levels (Figure 2a). These results indicate that UVB activates JNK and ERK1/2 MAPK in HPEKs. Taken together with the results shown in Figure 1, our analyses suggested that UVB irradiation results in the accumulation of ROS, which stimulate JNK and ERK1/2 MAPK activation.

### UVB-induced BNIP3 expression is mediated by JNK and ERK1/2 MAPK

Previous studies have reported that BNIP3 expression was regulated by JNK and ERK1/2 MAPK.^19^ Therefore, to determine whether the enhanced BNIP3 expression observed upon UVB irradiation is also mediated by JNK and ERK1/2, HPEKs were treated with the specific MAPK inhibitors, SP600125 and U0126, followed by UVB irradiation, and then subjected to western blot analysis. Both SP600125 and U0126 significantly reduced the UVB-induced expression of BNIP3 expression (Figure 2b) concomitant with an upregulation of cleaved caspase-3 (Figure 2b), suggesting that BNIP3 expression upon UVB irradiation has a crucial role in the protection of keratinocytes from apoptotic cell death. Consistent with these results, flow cytometry analysis of annexin-V-PI staining revealed that apoptosis was increased when HPEKs were treated with MAPK inhibitors (Figure 2e). As shown in Supplementary Figure S3, cell survival remained unaffected by MAPK inhibitors themselves without UVB treatment, suggesting that UVB-induced BNIP3 expression mediated by JNK and ERK1/2 MAPK was important for cell survival upon UVB exposure. In addition, organ-cultured skin from e14.5 mouse embryos revealed that apoptotic cell death was significantly increased upon UVB treatment when the skin was treated with SP600125 or U0126 compared with vehicle alone (Figure 2f). In addition, the autophagosome formation induced by UVB exposure was attenuated by SP600125 and U0126 (Figures 2b–d), suggesting that BNIP3-induced autophagy has a crucial role in the protection of HPEKs against UVB exposure. Furthermore, we noticed that the JNK inhibitor SP600125 inhibited not only JNK MAPK signaling as determined by the downregulation of phosphorylated c-jun, but also ERK1/2 MAPK signaling (Figure 2b). These data suggest that JNK MAPK regulates and cooperates with ERK1/2 MAPK to enhance the expression of BNIP3 in response to UVB irradiation.

### Autophagy is important to protect keratinocytes from UVB-induced apoptosis

**Figure 3:**
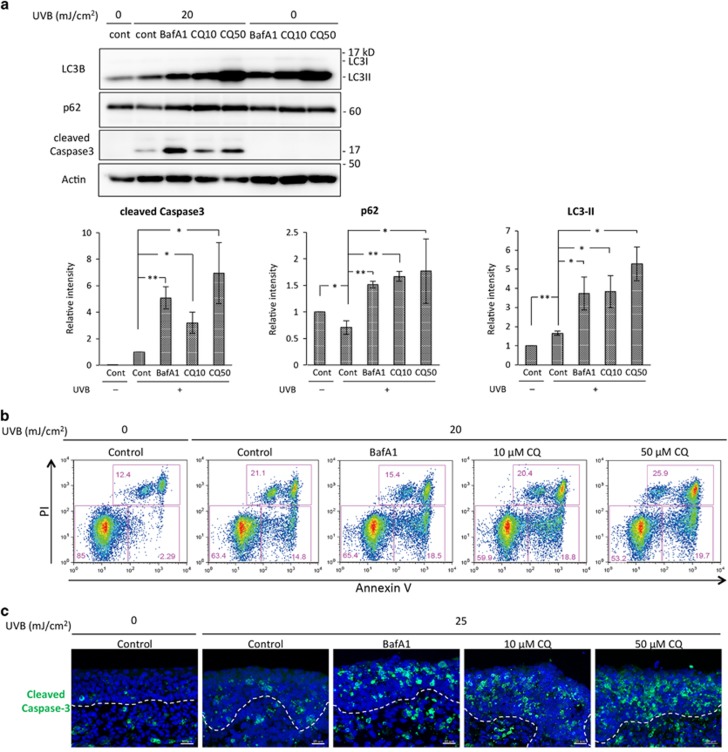
Autophagy is required for cell protection from UVB-induced apoptosis. (**a** and **b**) HPEK cells were exposed to UVB radiation (20 mJ/cm^2^) and incubated for 4 h. The autophagy inhibitors Bafilomycin A1 (Baf A1, 10 nM) and chloroquine (CQ, 10 or 50 *μ*M) were added 1 h prior to UVB exposure. (**a**) The extracted proteins were then immunoblotted with the indicated antibodies. Graphs indicate the relative band intensities as determined by ImageJ software and plotted as the means of seven independent experiments. **P<0.01. *P<0.05. Cont, control. (**b**) HPEKs were stained with annexin V-Alexa 488 and propidium iodide (PI). (**c**) The dorsal skin of a 14.5-dpc embryonic mouse was irradiated with 25 mJ/cm^2^ UVB, and cultured on Millicell culture inserts for 12 h. The autophagy inhibitors Bafilomycin A1 (Baf A1, 10 nM) and chloroquine (CQ, 10 or 50 *μ*M) were added 1 h before UVB exposure. After organ culture, the skin sections were processed for anti-cleaved caspase 3 immunostaining (green). The blue signals indicate nuclear staining. The dotted lines indicate the boundary between the epidermis and the dermis. Scale bars, 20 *μ*m

Our previous report demonstrated that the induction of BNIP3 expression upon UVB irradiation caused autophagy.^1^ In addition, our present study indicated that the downmodulation of UVB-induced BNIP3 expression by the antioxidant NAC or a MAPK inhibitor was concomitant with the attenuation of autophagosome formation (Figures 1b–d and 2b–d) and with the progression of cell death (Figures 1b, e, f, 2b, e and f). To investigate the involvement of autophagy in the protection of keratinocytes from UVB-induced apoptosis, the autophagy inhibitors bafilomycin A1 and chloroquine were added to HPEKs prior to UVB irradiation. As shown in Figure 3a, both compounds could successfully inhibit the degradation of LC3-II and p62 by autophagosomes. Notably, we could also observe a significant increment of the levels of cleaved caspase 3 in HPEKs treated with bafilomycin A1 and chloroquine, indicating that autophagy inhibition stimulated cell death by apoptosis upon UVB irradiation (Figure 3a). Furthermore, the numbers of apoptotic cells were significantly increased in HPEKs and organ-cultured skin from e14.5 mouse embryos following UVB irradiation upon prior treatment of the skin with autophagy inhibitors (Figures 3b and c). These data clearly indicate that the autophagy induction in response to UVB irradiation is indispensable for the protection of epidermal keratinocytes from UVB-induced apoptosis.

### BNIP3 degrades dysfunctional mitochondria in response to UVB exposure

**Figure 4:**
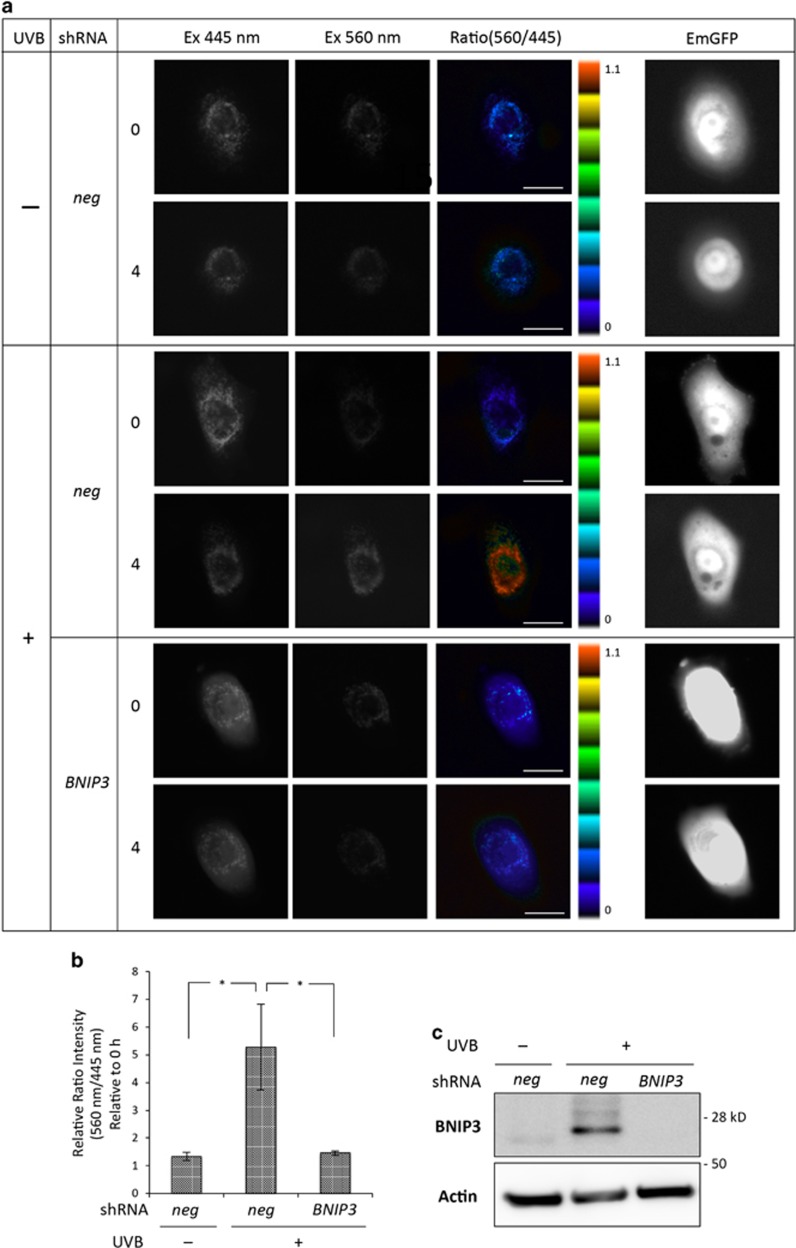
Mitophagy is induced by BNIP3 upon UVB irradiation. HPEK cells were transfected with plasmid vector expressing mitochondrial-targeted Keima-Red (mt-Keima) and shRNA with EmGFP as a marker. The cells were then exposed to UVB radiation (20 mJ/cm^2^) and incubated for 4 h. (**a**) Ratiometric imaging of mt-Keima imaging in HPEKs. Cells were imaged using a 445±45 nm or 560±25 nm excitation filter and a 641±75 nm emission filter. High ratio (560/445) signals, which originate from low pH compartments (lysosomes), are shown in red. Scale bars represent 20 *μ*m. (**b**) The graph indicates the relative signal ratio (560/445) intensities as determined by MetaMorph software. *N*=12–15 cells in each of three independent experiments. Error bars represent S.E. *P<0.05. (**c**) The proteins were extracted from the cells and then immunoblotted with the indicated antibodies

**Figure 5:**
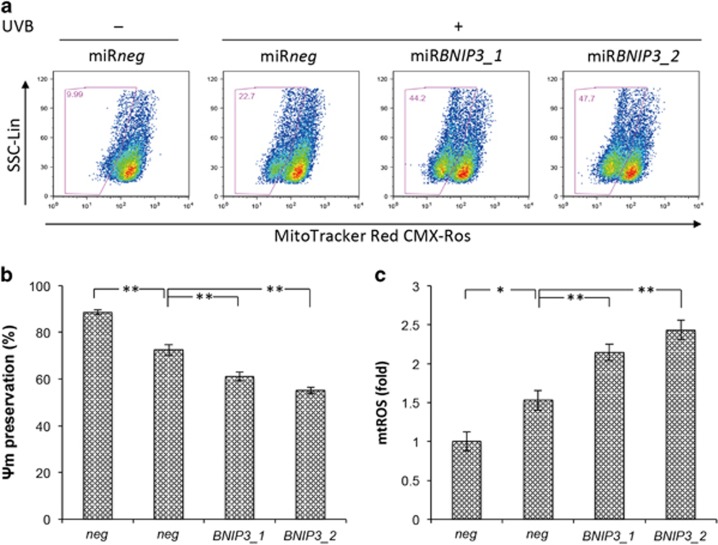
BNIP3 is required for the removal of dysfunctional mitochondria in epidermal keratinocytes after UVB exposure. HPEKs were infected with adenoviral vectors expressing shRNA followed by UVB radiation (20 mJ/cm^2^). (**a** and **b**) Mitochondrial membrane potential (ΔΨm) was measured using MitoTracker Red 4 h after UVB exposure. (**a**) Representative dot plots by flow cytometric analysis are shown. Gates represent cells with decreased ΔΨm. (**b**) Changes in ΔΨm were plotted as the means±S.E. of five independent experiments. **P<0.01. *P<0.05. (**c**) Mitochondrial ROS (mtROS) were measured using MitoSox Red. Graph indicates the quantification of mean fluorescence intensity (MFI) for MitoSox Red staining. Data are presented as the mean±S.E. from five independent experiments

**Figure 6:**
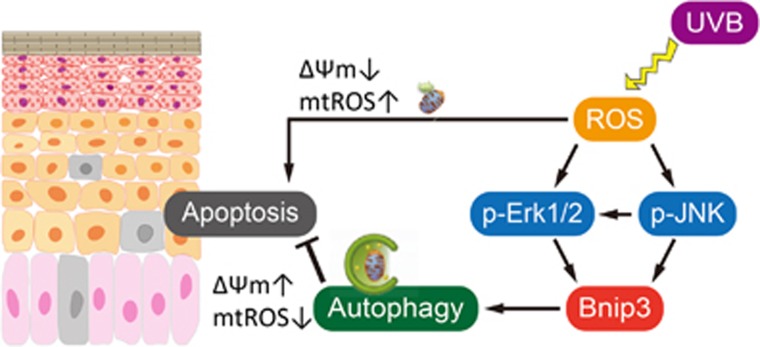
**Model of the role of BNIP3 in epidermal keratinocyte maintenance.** A schematic illustration of the results of this study is shown. UVB exposure triggers accumulation of ROS, which in turn activate ERK and JNK MAP kinase. Then, these MAP kinase activities stimulate the expression of BNIP3, which results in the induction of autophagy. Autophagy is required for the degradation of dysfunctional mitochondria, which is indispensable for the protection of keratinocytes from apoptosis

Our previous work has demonstrated that BNIP3 mediated the degradation of mitochondria during keratinocyte differentiation.^1^ To examine whether BNIP3 also degraded mitochondria in response to UVB exposure in HPEKs, a mitochondrial-targeted fluorescent protein mt-Keima that exhibits a reversible change in color in response to pH was monitored. mt-Keima has a bimodal excitation spectrum peaking at 440 and 586 nm corresponding to whether the protein is located in the cytoplasm (neutral environment) or the lysosome (acidic environment). Thus, a high mt-Keima signal ratio (560/445) indicates that mitochondria are being delivered to acidic lysosomes.^20^ Control keratinocytes did not generate high signal ratios for mt-Keima, indicating the absence of mitophagy. In contrast, the representative image shown in Figure 4a indicates that a large fraction of mitochondria was delivered to the lysosomes in response to UVB exposure. Conversely, BNIP3 knockdown significantly diminished the signal ratio intensities under UVB irradiation (Figures 4a and b). These data suggested that BNIP3 was required for the mitochondrial degradation by autophagy in response to UVB exposure. The knockdown efficiency of BNIP3 RNAi used in this study is shown in Figure 4c, Supplementary Figure S1 and in our previous report.^1^ Furthermore, we have analyzed the mitochondrial membrane potential (ΔΨm) and mitochondrial ROS (mtROS) levels, and found that mitochondria with lower ΔΨm and more mtROS were increased in HPEKs upon UVB exposure (Figures 5a–c). BNIP3 knockdown resulted in the further accumulation of mtROS (Figure 5c) and further decrease of ΔΨm (Figures 5a and b). These data demonstrated that BNIP3 was required for the removal of dysfunctional mitochondria. The overall model that we propose, based upon our findings, is shown in Figure 6.

## Discussion

BNIP3 has been reported to induce apoptosis, necrosis, or autophagy depending on the cellular context.^3^ In this study, we demonstrated that BNIP3 has a role in the maintenance of epidermal keratinocytes upon UVB irradiation through induction of autophagy (summarized in Figure 6). BNIP3 is indispensable for anti-apoptotic event upon UVB exposure, because BNIP3 knockdown resulted in the upregulation of cleaved caspase 3 and increase in Annexin-V^+^PI^-^ (early apoptotic) cell population (Supplementary Figure S1). This is consistent with previous suggestion of a role for autophagy in the skin epidermis;^1^ however, few attempts have been made to clarify this involvement.

The present data indicate that UVB upregulates BNIP3 expression, which is caused by intracellular ROS accumulation (Figure 1). ROS, which activate several signal transduction cascades, have been reported as a major mediator in the response to UVB irradiation. In particular, ROS have been reported to stabilize HIF-1*α*,^21^ which is a well-known inducer of BNIP3 transcription.^4, 7^ However, we detected neither stabilization of HIF-1*α* in response to UVB exposure nor downregulation of BNIP3 in HIF-1*α* knockdown cells, indicating that UVB-induced BNIP3 expression is independent of HIF-1*α* in HPEKs (Supplementary Figure S2). Another signaling candidate that is activated by ROS is MAPK, which is involved in directing cellular responses to a diverse array of stimuli. Several groups have reported that UVB irradiation induces the phosphorylation of EGFR, which in turn activates ERK1/2 and JNK MAPK pathways in keratinocytes.^13, 22, 23^ These reports also indicate that UVB-induced ROS act as mediators to activate MAPK. Consistent with these studies, our data also demonstrated that the activation of ERK1/2 and JNK MAPK in response to UVB exposure is mediated by UVB-induced ROS (Figure 1). Recently, it has been reported that Cadmium activates ERK and JNK MAPK, which in turn promote BNIP3 expression in neuronal cells.^19^ Notably, our data indicate that the upmodulation of BNIP3 in response to UVB is mediated by ERK1/2 and JNK MAPK (Figure 2). However, the mechanisms by which these MAPK pathways regulate BNIP3 expression remain unclear in the present study. Several researchers have elucidated the role of forkhead box (FOX) O3, a transcription factor that is activated by JNK, in BNIP3 expression.^24, 25^ The expression of BNIP3 has also been reported to be regulated by E2F-1,^26^ which is activated by Ras-induced ERK/MAPK.^27, 28^ Further studies are required to address these issues in HPEK cells and in response to UV irradiation.

Our data showed that ERK1/2 and JNK contributed to cell survival via BNIP3-induced autophagy under UVB-induced oxidative stress (Figure 2). The role of ERK1/2 and JNK activation in cell survival is cell-context dependent. Both pro-apoptotic and pro-survival roles have been attributed to these MAPKs.^29, 30^ However, the molecular mechanism by which JNK suppresses apoptosis is incompletely understood. Recently, several researchers have demonstrated that JNK is required for the cell survival via induction of autophagy,^31, 32, 33^ consistent with our present work. Our data also suggested that JNK activated ERK1/2 MAPK when HPEKs were irradiated with UVB, because phosphorylation of ERK1/2 was partially decreased by a JNK inhibitor (Figure 2). Specifically, the JNK inhibitor used in this study has been known to inhibit JNK1, -2, and -3 isoforms with similar potency but to exhibit greater than 300-fold selectivity against related MAPKs.^34^ In addition, we confirmed that the phosphorylation level of ERK1/2 was unaffected by this inhibitor in unirradiated HPEKs (Supplementary Figure S3). Furthermore, it has recently been reported that autophagy regulates ERK phosphorylation.^35^ Notably, our data indicated that autophagy inhibitors suppressed ERK phosphorylation (Supplementary Figure S4). Thus, our analyses suggest that JNK might regulate ERK phosphorylation via the induction of autophagy, which in turn contributes to cell survival; however, further analysis will be required to confirm this possibility.

Autophagy involves in both cell survival and cell death. In our present results, the significant enhancement of cell death by apoptosis following autophagy inhibition (Figure 3) indicates that BNIP3-induced autophagy is indispensable for cell survival. In addition to the autophagy, BNIP3 also appears to have dual roles in both autophagy-mediated survival and cell death. However, the means by which the cell switches between these different functions is not clear. Moreover, the mechanisms underlying how BNIP3-induced autophagy reduces UVB-induced apoptosis are still being clarified. One of the possible targets for autophagy in response to UVB is the mitochondrion. Our previous work has demonstrated that BNIP3 mediated the degradation of mitochondria during keratinocyte differentiation.^1^ Recent studies have also demonstrated that mitophagy is activated by BNIP3.^36, 37^ In particular, the present study indicated that BNIP3 was required for the induction of mitophagy in response to UVB (Figure 4). Damaged mitochondria are particularly prone to activating the apoptotic program by the release of pro-apoptotic factors, so their removal by autophagy can increase the threshold for apoptosis induction. O'Sullivan *et al.*have demonstrated that BNIP3- and BNIP3L-induced mitophagy is required for anti-viral natural killer cell survival through removal of mtROS.^38^ Our present data also represent an essential role of BNIP3 in the clearance of dysfunctional mitochondria and mitochondria-associated ROS (Figure 5). Therefore, the upregulation of BNIP3 by UVB exposure might be required for the protection of keratinocytes from UVB-induced apoptosis through mitochondrial degradation by autophagy. However, we could not exclude the possibility that denatured protein induced by UVB may be also degraded by BNIP3-induced autophagy. Accumulation of denatured protein may cause ER-stress, which results in the induction of apoptosis.^33, 39^ Further analysis will be required to confirm this hypothesis.

In summary, our data reveal that BNIP3-induced autophagy, which occurs via UVB-generated ROS-mediated JNK and ERK MAPK activation, is indispensable for the protection of keratinocytes from apoptosis, leading to the maintenance of skin homeostasis. Sun exposure is known to induce inflammation, barrier disruption, and the development of skin cancer. Our study thus might provide new insights into the functions of BNIP3 in the pathogenesis of UVB-induced skin disorders and highlight potential targets for therapeutic interventions.

## Materials and Methods

### Histology and immunofluorescence analysis

Samples were fixed in 4% paraformaldehyde, embedded in optimal cutting temperature (OCT) compound, frozen, and sectioned at 10 *μ*m. Sections were then subjected to immunohistochemical analysis as previously described.^40^ Briefly, the sections were stained with rabbit monoclonal antibody against cleaved Caspase3 (Asp175; Clone 5A1E; Cell Signaling Technology, Danvers, MA, USA), rabbit polyclonal antibody against GFP (ThermoFisher Scientific, Waltham, MA, USA), and mouse monoclonal antibody against LAMP2 (abcam, Cambridge, UK). For immunofluorescence analysis using the LC3 antibody (MBL, Nagoya, Japan), the cells were permeabilized with PBS containing 100 *μ*g/ml Digitonin (Sigma-Aldrich, Saint Louis, MO, USA) instead of PBST (PBS, 0.5% Triton X-100). Images were obtained using a fluorescence microscope (BZ-9000; Keyence, Osaka, Japan) and were analyzed using BZ Analyzer Software (Keyence). For the quantitation of autophagosomes, images with at least 100 representative cells per condition in three independent experiments were analyzed using IN Cell Investigator software (GE Healthcare, Chicago, IL, USA).

### Cell culture

HPEKs were purchased from CELLnTEC (Bern, Switzerland) and maintained in CnT-PR (CELLnTEC) culture medium according to the manufacturer's protocol.

### Adenovirus and lentivirus infection

Adenoviruses expressing shRNA against *BNIP3* were constructed using the ViraPower adenoviral expression system (ThermoFisher Scientific) according to the manufacturer's protocol. Lentivirus expressing EGFP-LC3 (from Addgene plasmid 21073, Cambridge, MA, USA) was constructed and used to infect keratinocytes as previously described.^1^

### Skin organ culture

Skin organ culture was performed as previously described.^40^ Skin specimens were prepared from the dorsal skin of E14.5 embryos derived from C57BL/6 mice (SLC, Inc., Kyoto, Japan) and cultured in DMEM supplemented with 10% fetal bovine serum. All animal experiments were performed in accordance with the guidelines of the National Institutes of Health Guide for the Use of Laboratory Animals and were approved by the Animal Research Committees of Kindai University.

### Western blot analysis

Cells were lysed with lysis buffer (20 mM Tris-HCl (pH8.0), 1% SDS, and 1 mM DTT). Blots were probed with a mouse monoclonal antibody against BNIP3 (Clone ANa40; Abcam; ab10433), rabbit monoclonal antibody against cleaved Caspase3 (Asp175; Clone 5A1E; Cell Signaling Technology; #9664), rabbit monoclonal antibody against SAPK/JNK (Clone 56G8; Cell Signaling Technology; #9258), rabbit monoclonal antibody against phospho SAPK/JNK (T183/Y185; Clone 81E11; Cell Signaling Technology; #4668), rabbit polyclonal antibody against phospho c-Jun (S63; Cell Signaling Technology; #9261), rabbit monoclonal antibody against p44/42 MAPK (Erk1/2; Clone 137F5; Cell Signaling Technology; #4695), rabbit monoclonal antibody against phospho p44/42 MAPK (T201/Y204) (Clone D13.14.4E) (Cell Signaling Technology; #4370), rabbit polyclonal antibody against LC3B (Cell Signaling Technology; #2775), rabbit polyclonal antibody against p62 (MBL; PM045), and mouse monoclonal antibody against Actin (Clone C4) (Merck-Millipore, Billerica, MA, USA; MAB1501). Horseradish peroxidase (HRP)-conjugated anti-mouse or rabbit IgG secondary antibody (Cell Signaling Technology; #7076, #7074) was used as a probe, and immunoreactive bands were visualized with the Immobilon Western Chemiluminescent HRP substrate (Merck-Millipore). The band intensity was measured using ImageJ software (NIH, Bethesda, MD, USA).

### UVB irradiation

Prior to UVB irradiation, the cells and the cultured skin were washed with PBS and irradiated with a handheld UV Lamp (UVP, Upland, CA, USA). The accuracy of the UVB dose was calculated using a UVX-31 Radiometer (UVP).

### Measurement of intracellular ROS levels

Prior to measurement of intracellular ROS, the cells were cultured in the absence or presence of 5 mM NAC for 1 h. The cells were then loaded with 5 *μ*M CM-H_2_DCFDA for 30 min and exposed to 20 mJ/cm^2^ UVB, followed by lysis with 0.1% Triton X-100 at 10 min after exposure. The fluorescence intensity of each lysate was measured with an excitation of 485 nm and emission at 528 nm using a Synergy H4 microplate reader (BioTeK, Winooski, VT, USA). Intracellular ROS levels are expressed as the fold change of the fluorescence intensity.

### Annexin V-PI staining

Apoptosis was measured by flow cytometry after staining with Alexa Flour 488 Annexin V and Propidium Iodide (PI) using Alexa Fluor 488 Annexin V/Dead Cell Apoptosis Kit (ThermoFisher Scientific). The staining was performed according to the manufacturer's instructions. For each sample, at least 1 × 10^4^ cells were analyzed by flow cytometry (ec800 cell analyzer, SONY, Tokyo, Japan). FlowJo (TreeStar Inc., Ashland, OR, USA) software was used for quantitative analysis.

### Measurement of mitochondrial membrane potential and mitochondrial ROS

Mitochondrial membrane potential (ΔΨm) was measured using MitoTracker Red (ThermoFisher Scientific) following the manufacturer's instructions. Mitochondrial ROS were measured using MitoSox Red (ThermoFisher Sicentific). The staining was performed according to the manufacturer's instructions. For each sample, at least 1 × 10^4^ cells were analyzed by flow cytometry (ec800 cell analyzer, SONY). FlowJo (TreeStar) software was used for quantitative analysis.

### Quantification of mitophagy

For quantifying mitophagy, HPEKs were transiently transfected with pMitophagy-mKeima-Red mPark2 (MBL)^20^ using the Amaxa nucleofector 4D system (Lonza, Walkersville, MD, USA). Briefly, 2 × 10^6^ cells were resuspended in 100 *μ*l Amaxa nucleofector solution (Primary nucleofector kit P3) and mixed with 1 *μ*g pMitophagy-mKeima-Red mPark2 and 1 *μ*g pcDNA6.2/ EmGFP-miRneg or pcDNA6.2/EmGFP-miRBNIP3^1^ for each sample. Electroporation was performed using nucleofection (Amaxa DT-138 nucleofector program). Ratio (560/445) images of mitochondrial-targeted Keima-Red were created and analyzed using MetaMorph 7.8 software (Molecular Devices, Sunnyvale, CA, USA). Ratio values ranged from 0 to 1.1 at all times.

### Statistical analysis

Statistical differences were evaluated with a Student's *t*-test or a ratio t-test. A value of *P*<0.05 was considered statistically significant (**P<0.01, *P<0.05). All statistical tests were two-sided.
