# Supplementary figures and images for: BNIP3 upregulation via stimulation of ERK and JNK activity is required for the protection of keratinocytes from UVB-induced apoptosis
Source: Cell Death Dis. 2017 Feb 2;8(2):e2576–. doi: 10.1038/cddis.2017.4 (PMC5386491; doi:10.1038/cddis.2017.4)

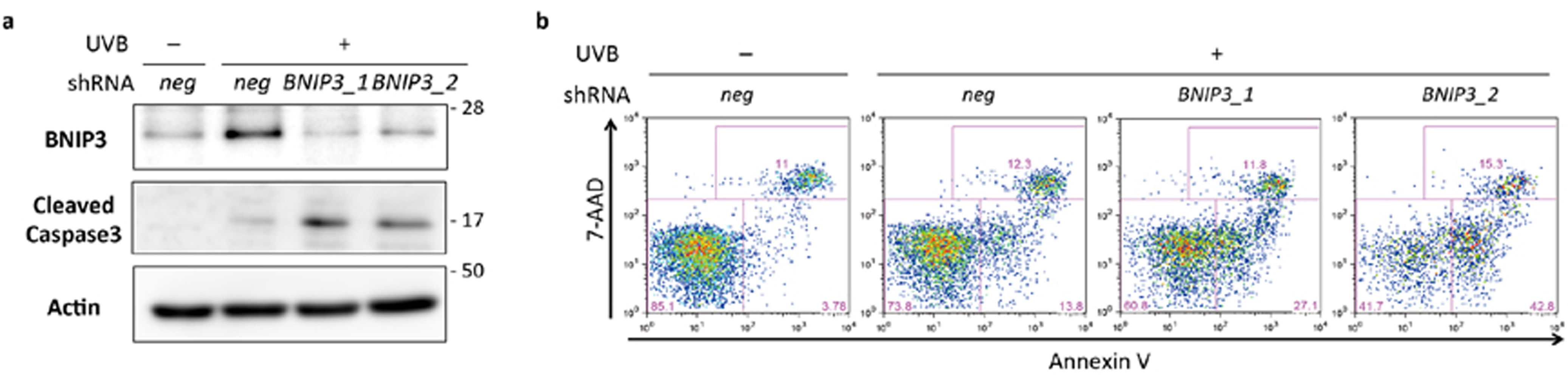

Supplement: Supplementary Figure 1 [file cddis20174x1.tif]

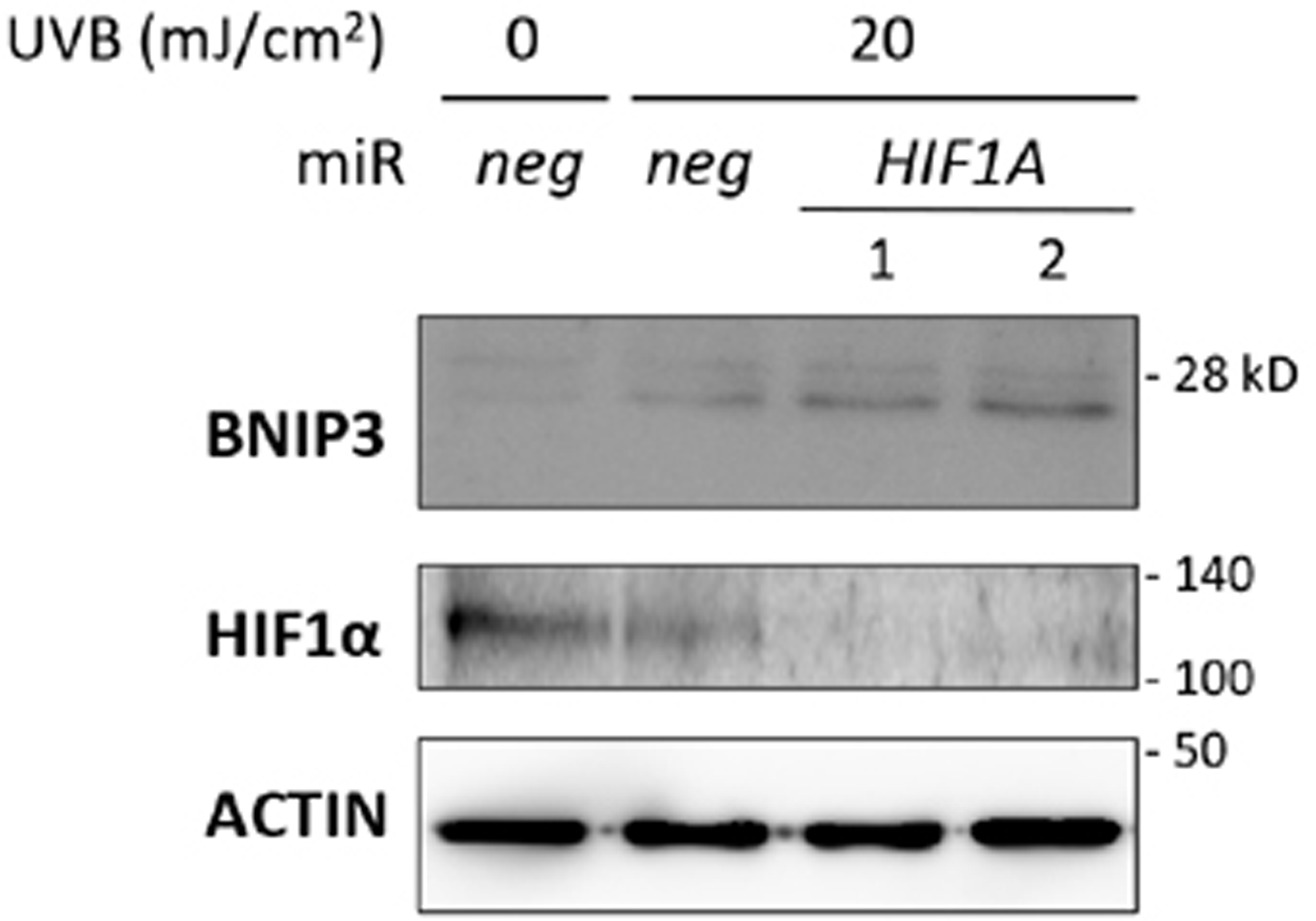

Supplement: Supplementary Figure 2 [file cddis20174x2.tif]

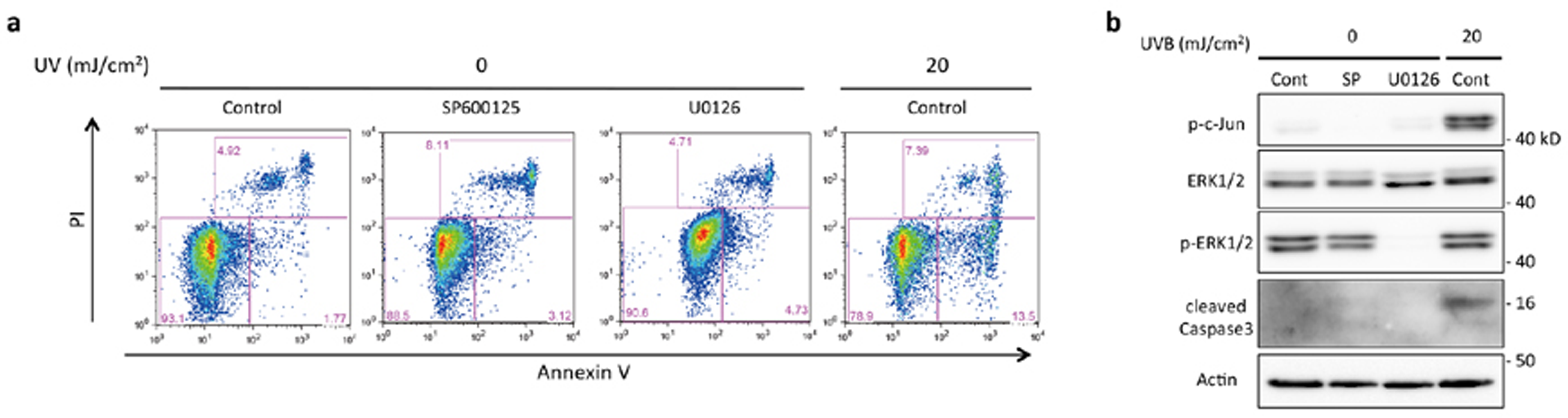

Supplement: Supplementary Figure 3 [file cddis20174x3.tif]

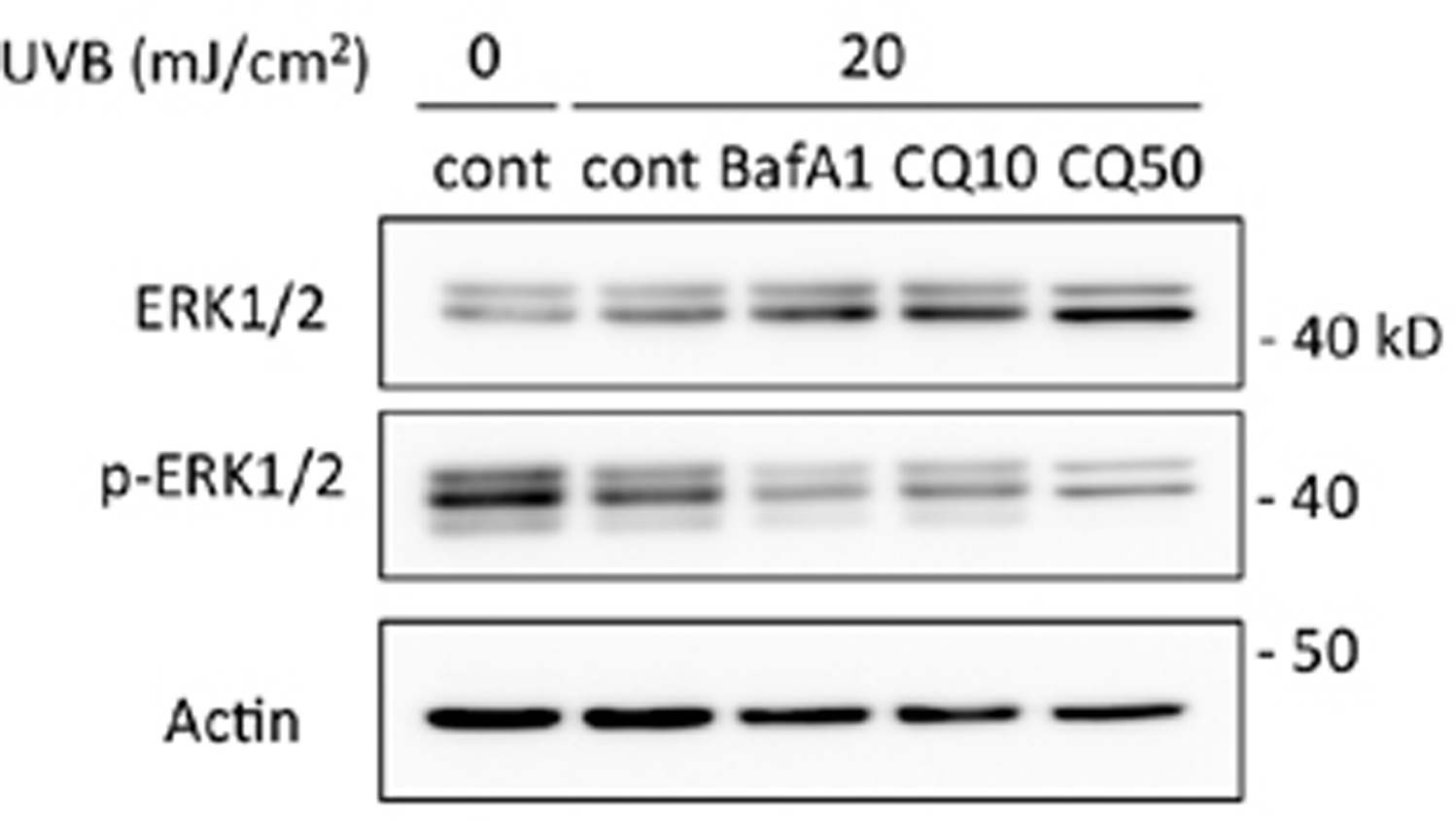

Supplement: Supplementary Figure 4 [file cddis20174x4.tif]
