# Supplementary material for: BNIP3 upregulation via stimulation of ERK and JNK activity is required for the protection of keratinocytes from UVB-induced apoptosis
Source: Cell Death Dis. 2017 Feb 2;8(2):e2576–. doi: 10.1038/cddis.2017.4 (PMC5386491; doi:10.1038/cddis.2017.4)
Supplement: Supplementary Information [file cddis20174x5.docx]

**Supplemental Materials**

**Adenovirus**

Adenovirus expressing miR*HIF1A* was generated as previously described (ref. 1). Briefly, oligonucleotides targeting a human *HIF1A* sequence compatible for use in cloning into BLOCK-iT Pol II miR RNAi expression vectors (Invitrogen) were obtained using the online tool BLOCK-iT RNAi Designer. The oligonucleotide sequences used in this study are: miR*HIF1A*_1_top: TGCTGATCAGCACCAAGCAGGTCATAGTTTTGGCCACTGACTGACTATG

ACCTTTGGTGCTGAT, miR*HIF1A*_1_bottom: CCTGATCAGCACCAAAGGTCATAGTCAGTCA

GTGGCCAAAACTATGACCTGCTTGGTGCTGATC, miR*HIF1A*_2_top: TGCTGTAAAGCATCA

GGTTCCTTCTTGTTTTGGCCACTGACTGACAAGAAGGACTGATGCTTTA, miR*HIF1A*_2_bottom: CCTGTAAAGCATCAGTCCTTCTTGTCAGTCAGTGGCCAAAACAAGAA

GGAACCTGATGCTTTAC. Cloning procedures were performed following the manufacture’s instructions.

**Antibody**

A mouse monoclonal antibody against HIF-1α (Clone H1alpha67) was purchased from Novus Biologicals (Denton, TX, USA).

1. Moriyama M, Moriyama H, Uda J, Matsuyama A, Osawa M, Hayakawa T. BNIP3 plays crucial roles in the differentiation and maintenance of epidermal keratinocytes. *J Invest Dermatol*. 2014;134(6):1627-35.

**Supplemental Figures**

**Figure S1. BNIP3 protects HPEKs from UVB-induced apoptosis. (a, b)** HPEKs were infected with adenovirus expressing shRNA against BNIP3 and irradiated with UVB. Cells were subjected to western blot analysis **(a)** or flow cytometry analysis for annexin V/7-AAD labeling **(b)** 8 h after irradiation.

**Figure S2. BNIP3 expression is not regulated by HIF-1α.** HPEKs were infected with adenovirus expressing miR *neg*, miR *HIF1A_1* or miR *HIF1A_2*, and irradiated with UVB. Cells were subjected to western blot analysis 8 h after irradiation.

**Figure S3. Cell survival remains unaffected by MAPK inhibitors without UVB treatment. (a, b)** HPEK cells were treated with 0.1 % DMSO (Control), 10 µM SP600125, and 10 µM U0126 for 9 h without UVB treatment. DMSO was added 1 h before UVB exposure (20 mJ/cm^2^) followed by 8 h incubation. Then the cells were subjected to flow cytometry analysis for annexin V/PI labeling **(a)** or western blot analysis **(b).**

**Figure S4. Autophagy activates ERK1/2 MAPK.** HPEKs were exposed to UVB radiation (20 mJ/cm^2^) and incubated for 4 h. Autophagy inhibitors, 10 nM of Bafilomycin A1 (Baf A1) and 10, 50 µM of chloroquine (CQ) were added 1 h before UVB exposure. The extracted proteins were then immunoblotted with the indicated antibodies.
